# Development of a pyrosequencing assay for the typing of alphaherpesviruses^☆^

**DOI:** 10.1016/j.mex.2015.01.001

**Published:** 2015-01-12

**Authors:** G. Fusco, M.G. Amoroso, N. Gesualdi Montesano, M. Viscardi

**Affiliations:** a Department of Animal Health, Experimental Zooprophylactic Institute of Southern Italy, Via Salute, 2, 80055 Portici (NA), Italy; b Qiagen Srl, Via Grosio 10/8, 20151 Milano, Italy

**Keywords:** Typing of alphaherpesviruses by pyrosequencing, Pyrosequencing, Alphaherpesviruses, Polymorphic site, PyroMark, Biotinylated primer, US8 gene

## Abstract

Identification of herpesvirus in biological material is usually carried out by real-time PCR. With the aim to classify the strain of virus identified, real-time PCR must be often supported by time-consuming capillary electrophoresis sequencing analysis. Here we provide a protocol for the rapid and reliable identification of 5 closely related herpesviruses by PyroMark Q24 sequencing system. PyroMark performs DNA sequencing analysis using pyrosequencing, a technology based on the detection of released pyrophosphate during DNA elongation [1]. PyroMark is designed to detect changes in specified variable positions of the DNA. It can efficiently detect single nucleotide differences in sequences [2]. In the present paper we describe a protocol to pyrosequence a small polymorphic segment of the US8 gene. On the basis of the differences identified in the nucleotide sequence we could readily classify the herpesvirus as *Bovine herpesvirus 1.1, Bovine herpesvirus 1.2, Bovine herpesvirus 5*, *Bubaline herpesvirus 1* or *Caprine herpesvirus.* The protocol set up offers several advantages with respect to the techniques commonly used:

• it requires less than one working day to be carried;
• it gives the possibility to analyze, at reasonable costs, up to 24 samples at a time; and
• it allows to detect with great reliability and specificity strongly genetically correlated organisms like the herpesviruses named above. The procedure can be easily applied to other families of viruses, with opportune modifications.

## Method details

In short the typing of the alphaherpesviruses by pyrosequencing is made as follows: (A) extraction of DNA from the samples; (B) PCR amplification with two amplification primers (forward and reverse) that are specific to the sequence of interest, one of which (in our case the reverse primer) is biotinylated; (C) capture of biotin-labeled single-stranded PCR products (ssDNA) and separation from non biotin-labeled strands; (D) incubation of the ssDNA with the sequencing primer; (E) sequencing of the variable region by pyrosequencing; (F) analysis of the pyrograms obtained and classification of the strain of herpesvirus.

## Experimental design

**Fig. 1:**
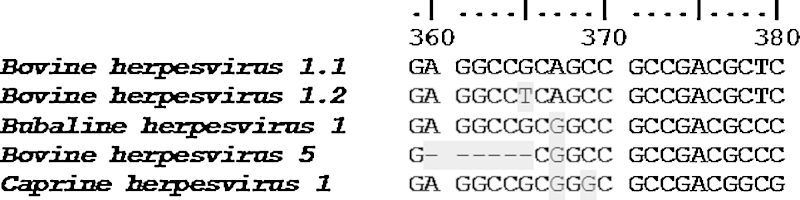
ClustalW alignment of the polymorphic site of the 5Herpesvirus US8 sequences. In grey the nucleotide differences with respect to *Bovine herpesvirus 1.1*. The alignment was carried out with the Bioedit sequence alignment editor software.

**Fig. 2:**
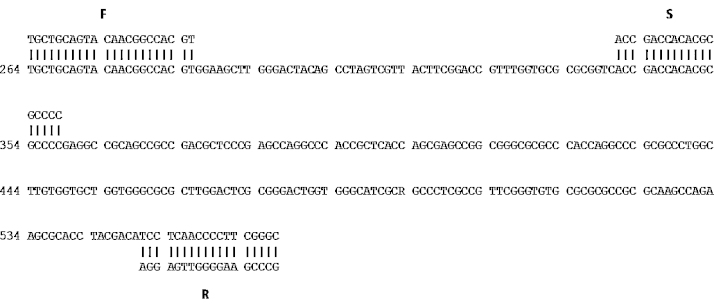
Schematic representation of the pyrosequencing assay with forward (F), reverse (R) and sequencing (S) primers. The sequence of DNA refers to a region (302 pb) of the gene US8 of the *Bovine herpesvirus* 1.1.

**Fig. 3:**
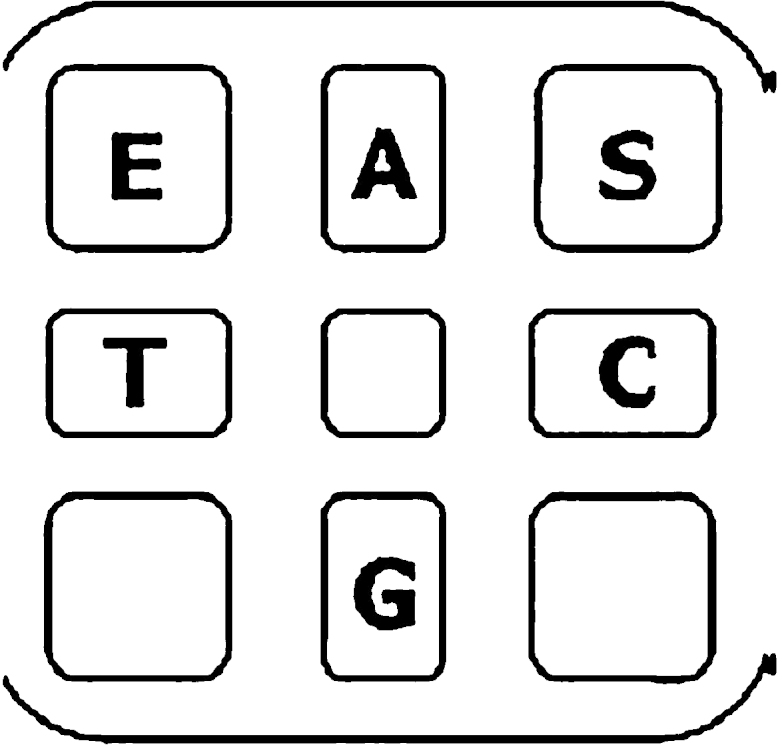
PyroMark Q24 cartridge scheme of loading. The amount of reagents to add per sample is the following: enzyme mix (E): 54 μl; substrate mix (S) 54 μl, nucleotide A (A) 52 μl, nucleotide C (C) 52 μl, nucleotide G (G) 52 μl, nucleotide T (T) 52 μl.

Pyrosequencing assay involves 3 primers: forward, reverse and sequencing. To design the primers we first performed a multiple alignment (ClustalW alignment software) of the US8 gene of the 5 herpesviruses (Fig. 1) and, on the basis of the alignment, we built up the pyrosequencing assay (Fig. 2). As it is possible to see from Fig. 2, forward and reverse primers has been designed to amplify an amplicon (302 bp) common to all the herpesviruses in study and containing a short sequence variable among the strains (polymorphic site). The sequencing primer was designed to be located just upstream the polymorphic region in order to analyze downstream nucleotide segment. All the primers were designed with the PyroMark Assay Design Software 2.0 (Qiagen) setting the assay type as “sequence analysis (SQA)”. After primers design, with the help of the PyroMark Q24 2.0.6 software we built up the pyrosequencing running protocol (PRP), establishing the set up of the plate and the PyroMark SQA assay. The software, on the basis of the information filled in (including the number of samples to analyze), supplies the total volume of reagents to add to the cartridge (see Fig. 3) and the order of cyclic nucleotides dispensation (8x[ACGT]). All the assay information is saved on an electronic key and transferred to the PyroMArk Q24 station where the assay is run.

### Step 1: nucleic acid extraction

#### Materials

##### Viral strains used to validate the method

Reference strains of *BoHV-1.1*, *BoHV-1.2*, *BoHV-5* and *CpHV-1* were supplied by Dr. M. Beer from FLI (Friedrich Loeffler Institut, Germany). *Bubaline herpesvirus* 1 (strain Metzler) was kindly provided by Prof. U. Pagnini (University of Naples Federico II, Italy).

• 3–7 mm stainless steel beads
• Disposable scalpel
• Sterile pincers and scissors
• QIAamp viral RNA mini kit (Qiagen)
• Technical scale
• Tissue Lyser (Qiagen)

The protocol is described for animal tissues but can be easily applied to samples like plasma, urine, cell culture, swabs and blood. It is possible to work with lower/higher amount of tissue paying attention to maintain the ratio 1:10 tissue/PBS. If processing fiber-rich tissues, complete disruption and homogenization may sometimes not be possible. However, small amounts of debris have no effect on subsequent nucleic acid extraction.

#### Procedure

• Cut the tissue of each sample under analysis in small pieces with the help of the scalpel, the pincers and the scissors.
• Weight 150 mg of tissue and place it in a 2 ml microcentrifuge tube.
• Add 1 stainless steel bead (3–7 mm mean diameter) and 1.5 ml of PBS.
• Place the tubes in the TissueLyser and omogenize for 5 min at 30 Hz.
• The duration of disruption and homogenization depends however on the tissue being processed and can be extended until no tissue debris is visible.
• Centrifuge the samples 15 min/4000 × *g*/4 °C.
• Transfer the supernatant in a new 2 ml microcentrifuge tube.
• Process 140 μl of the supernatant with QIAamp viral RNA mini kit following handbook instructions.
• Elute nucleic acids in 50 μl of QIAamp elution buffer.
• Amplify immediately extracted DNA or store it at −20 °C until use.

### Step 2: PCR amplification

#### Materials

• Forward specific primer: 5′-TGCTGCAGTACAACGGCCACGT-3′;
• Reverse biotinylated specific primer: 5′-GCCCGAAGGGGTTGAGGA-3′.
• PyroMark PCR master mix (Qiagen)
• Qsolution
• PCR amplification system
• Capillary electrophoresis system

#### Procedure

• Prepare, in a 1.5 ml microcentrifuge tube, the PCR reaction mix including per sample: 12.5 μl PyroMark PCR master mix, 1.25 μl of each primer (0.2 μM), 5 μl Qsolution.
• Distribute the mix (20 μl/sample) in 0.2 ml PCR tubes
• Add 5 μl of sample (extracted DNA) to the relative tube.
• Perform PCR amplification with the following thermal profile: a first activation step of 15 min at 95 °C, 45 cycles of: 30 s at 94 °C, 30 s at 60 °C, and 30 s at 72 °C and a final elongation step of 10 min at 72 °C.
• Check PCR product by capillary electrophoresis. Conventional agarose gel electrophoresis can be carried out if capillary electrophoresis system is not available. *Note*: if non specific amplicons also result from the PCR it is necessary to perform further optimization of the PCR conditions.

### Step 3: preparation of the samples and pyrosequencing

#### Materials

• PyroMark Q24 Vacuum workstation (Qiagen)
• Plate mixer for immobilization to beads
• Heating block capable of attaining 80 °C
• PyroMark Q24 plate (Qiagen)
• PyroMark Plate Holder (Qiagen)
• PyroMark Q24Cartridge (Qiagen)
• Streptavidin Sepharose High Performance (GE Healthcare)
• Sequencing specific primer: 5′-ACCGACCACACGCGCCCC-3′
• PyroMark Binding buffer
• PyroMark Denaturation solution
• PyroMark Wash buffer concentrate
• PyroMark Annealing buffer
• PyroMark Gold Q24 reagents (containing enzymes, substrates and nucleotides for pyrosequencing)

#### Procedure

**Fig. 4:**
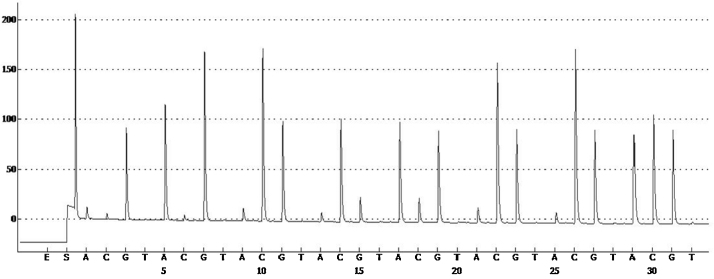
SQA pyrogram report run. Example of the pyrogram resulting from *Bubaline Herpesvirus 1.* Sequence read: GAGGCCGCAG CCGCCGACG.

• Prepare, in a 2 ml microcentrifuge tube, the immobilization solution by mixing (per sample): 2 μl steptavidin-coated Sepharose beads together with 40 μl Binding buffer and 18 μl high-purity water.
• Transfer biotinylated amplified samples (20 μl) to the wells of a PCR plate.
• Add 60 μl of immobilization solution to the samples in the PCR plate.
• Seal the plate and capture PCR products on the steptavidin-coated beads by shaking the plate on a plate mixer for 10 min at 1400 rpm.
• In the meanwhile prepare the vacuum workstation by filling the 5 troughs with the following solutions:
  1) 50 ml ethanol 70%
  2) 40 ml Denaturation solution
  3) 50 ml 1x Washing buffer
  4) 50 ml high-purity water
  5) 70 ml high-purity water
• Immediately after immobilization perform separation of biotinylated PCR strands from non-biotinylated strands in the vacuum workstation as indicated in the PyroMark user manual. *Note*: Beads sediment quickly, so be careful to perform this step right away after the capture.
• At the end of the procedure samples will be realized into a PyroMArk Q24 (PMQ) plate which contains 25 μl (per well) of the sequencing primer (opportunely diluted to 0.3 μM in the Annealing buffer).
• Accommodate the PMQ plate into the PyroMark Plate Holder
• Perform annealing of the primer to the samples by heating the PyroMark plate at 80 °C for 2 min and cooling it for 5 min at room temperature.
• Prepare the Gold Q24 reagents (enzyme and substrate mixtures) as indicated by the manufacturer.
• Select the PRP in the PyroMark Q24 2.0.6 software and set the number of samples and the scheme of the PMQ plate.
• Transfer, with the help of the electronic key, the assay information on the PyroMark Q24 station.
• Pipet the PRP established amount of reagents into the cartridge compartments according to Fig. 3.
• Place the PMQ plate and the cartridge in the instrument and start the run.
• Analyze obtained pyrograms (Fig. 4) with the PMQ24 sequencing software (Qiagen).
• Classify the herpesvirus comparing the sequence obtained to those expected for each strain. *Note:* The lists of materials include only non standard items. General materials like microcentrifuge tubes, or PBS are assumed to be available. For PCR and pyrosequencing steps it is recommended to use only PCR clean disposable items.

## Additional information

### Background

The PyroMark Q24 system uses pyrosequencing technology for real-time, sequence-based detection of sequence variants, being able to analyze segments of DNA up to 100 nucleotides in length [3], [4]*.* Pyrosequencing technique has already been successfully employed for the typing of herpes simplex virus types 1 and 2 [3], as well as human papillomavirus [5]. During Pyrosequencing reaction the sequencing primer is extended to provide the sequence of the template DNA. Nucleotides are added one at a time in a predefined order. The first nucleotide is added and if it is complementary to the base in the template strand it will be incorporated into the elongating DNA strand by DNA polymerase. The incorporation is accompanied by release of pyrophosphate (PPi). PPi is converted to ATP by ATP sulfurylase, in the presence of adenosine 5′ phosphosulfate. This reaction catalyses the conversion of luciferin to oxyluciferin by luciferase, with the emission of visible light that is recorded by a LCC camera. The complementary sequence is built up and each nucleotide incorporation generates light seen as a peak in the Pyrogram. The sequence is determined, at the end of the run, from the peaks in the Pyrogram.
